# General Hospital of Vienna

**Published:** 1875-03-01

**Authors:** Lyman Ware

**Affiliations:** Vienna


### GENERAL HOSPITAL OF VIENNA.

EDITORS MEDICAL EXAMINER: The Vienna General Hospital has as usual been filled to its utmost capacity during the winter, and as it contains about four thousand beds, we have had no want of interesting material. The out-door department, the ambulatory service, also furnishes an almost infinite variety and number of patients, particularly in the Eye, Ear and Skin departments; and perhaps I might also add, the Laryngeal department, which, since the time of Turck, is a most important branch in the hospital, and in which several hundred are daily treated. There is scarcely a doubt but that in hospital experience Vienna surpasses the world. The method of instruction is in perfect accord with that which you have so long insisted upon, viz.: bedside observation and examination. Notwithstanding there are a thousand students, there are so many lecture-rooms, and they are arranged in such a manner that each student has abundant opportunity for special examination. Such is particularly the case in the Laryngeal and Ophthalmological wards, where each student has his own table and light, and makes his examinations and applications under the immediate direction of the Professor or one of his assistants. Each lecture continues from one to two hours, half of the time being spent in examinations, the other half in explanations by the Professor. Hebra, whose name is almost a household word the world over, is as active and interesting as ever. He is punctually at his post every morning at eight o'clock, and has been absent from his clinic but once or twice the entire winter. The last volume of his work on skin diseases is recently published, but owing to its high price, twenty-five dollars (50 fl.), it will not have a large circulation, as the general practitioner and student will prefer some less voluminous work.

The method adopted by Gruber for aural instruction is most excellent. Aside from several large wards, he has a great many out-door patients who come regularly to be inspected and treated by the class, of course under his direct supervision. Each patient is numbered, and a corresponding number with the characteristic appearance of the *membrani tympani*, is placed upon a blackboard, so that each student cannot fail to fully comprehend the pathological change. In this manner twenty to thirty cases are daily examined. Students are daily called upon to make a diagnosis, and also give explanations of abnormal appearances. Prof. Gruber has recently divined a suction syringe, for the purpose of draining pus from the middle ear, which he considers of the greatest value to the aural surgeon.

He says by means of the air balloon alone it is impossible to empty the middle ear of pus, even if the membrane should contain a large perforation, which is by no means always the case, as the pus is more or less tenacious, and lies at the bottom of the chamber. Then, too, there is always danger of driving the pus into the mastoid cells, where it must excite further inflammation. By the timely use of the instrument, he thinks inflammation of the mastoid cells can often be averted. He generally uses the instrument with the head mirror, so that he can have both hands quite free, the one to use the instrument, the other to adjust the speculum. Scarcely a day passes that he does not demonstrate the value of the instrument and the skill with which he makes use of it.

Much more attention is given to the microscope here than with us. With us students are obliged to understand practical anatomy, and why not also histology? How can they comprehend histology without practical or personal work with the microscope?

Professor Schenk, formerly assistant to Brucke, now professor of Embryology, is the great favorite with American students here who are devoting any time to microscopy, as most of them are. His laboratory, which can accommodate from forty to fifty students, is open from 8 o'clock in the morning until 12 at night, not excepting even Sundays, so that one can easily select the most convenient hours for work. The Professor is untiring in his attention to the students, and is in the laboratory from 9 a. m. until late in the afternoon. Although a young man, he has already created for himself a European reputation in Embryology. Yet, with all his skill, in the earlier stages of embryonic life he is unable to determine the higher from the lower forms of animals, Man and Monkey being quite the same.

LYMAN WARE, M.D.

VIENNA, Jan. 15th, 1875.

---

HYPODERMIC INJECTION OF ERGOT IN POST-PARTUM HÆMORRHAGE.—Dr. P. C. Williams, of Baltimore, in a paper read before the Medico-Chirurgical Faculty of Maryland, 1874, recommends the hypodermic injection of the fluid extract of ergot in post-partum hæmorrhage. He gives in detail three cases, in which he has found its use most advantageous in stopping severe flooding. In two of the cases it was not tried until other remedies had been employed to no purpose. The effect of the hypodermic was almost instantaneous, and it was permanent. The fluid was injected in the inside of the thigh, and in no case was an abscess produced. —*Obstetrical Journal.*

